# Positive communication workshops: are they useful for treatment programmes for anorexia nervosa?

**DOI:** 10.3389/fpsyg.2023.1234928

**Published:** 2023-08-14

**Authors:** Kate Tchanturia, Philippa Croft, Victoria Holetic, Jessica Webb, Marcela Marin Dapelo

**Affiliations:** ^1^Department of Psychological Medicine, Institute of Psychiatry, Psychology and Neuroscience, King’s College London, London, United Kingdom; ^2^Eating Disorders National Service, South London and Maudsley NHS Foundation Trust, London, United Kingdom; ^3^Department of Psychology, Ilia State University, Tbilisi, Georgia

**Keywords:** eating disorders, group, positive psychology, communication, brief therapy, treatment

## Abstract

**Background:**

Social isolation, loneliness and difficulties in relationships are often described as a core feature of eating disorders. Based on the experimental research, we have designed one-off workshops for patients in inpatients and day care services and evaluated its acceptability and effectiveness using feedback questionnaires.

**Methods:**

This naturalistic project is an evaluation of multiple positive communication workshops. Forty-one participants completed workshop questionnaires, which were provided immediately at the beginning and end of the workshop, including feedback on these one-off groups. The workshops consisted of educational and experiential components. The questionnaire outcomes were evaluated by independent researchers.

**Results:**

All participants were female adults with a mean age of 33 (12.2) and a diagnosis of Anorexia Nervosa (AN; either restrictive or binge-purge subtype). Post-workshop questionnaires showed large effect sizes in the improvement of understanding the importance and confidence in using positive communication strategies.

**Discussion:**

Addressing social communication difficulties in eating disorder treatment programmes adds valuable dimensions to these symptom-based treatments in both inpatient settings and day services, and may provide broader benefits in overall social functioning in patients with AN.

**Conclusion:**

Brief one-off workshops targeting social functioning for patients with eating disorders might be useful complementary input for treatment programmes.

## 1. Introduction

Anorexia Nervosa (AN) is a psychiatric condition characterized by both physical health difficulties, such as low weight through intake restriction, and mental health difficulties, such as body image disturbances (American Psychiatric Association, 2013). Significant social and functional difficulties are also described as core features of AN (Harrison et al., 2014). Due to the high physical and psychological risk attached to severe eating disorders (ED), professional clinical teams are often occupied with supporting individuals with their nutritional needs in intensive care programmes (i.e., inpatient and day services), through brief and symptom-targeting psychological therapies.

Difficulties in social and emotional processing are recognized as a maintaining factor for AN (Treasure and Schmidt, 2013). Previous studies have proposed that individuals with AN experience emotions to be threatening, meaning when emotions arise, they tend to engage in fasting to avoid them; thus, the symptomatic behaviour acts as a maladaptive means to regulate emotions [e.g., (Wildes et al., 2010; Treasure and Schmidt, 2013; Lynch et al., 2015)]. Studies with self-reported methodologies have also demonstrated that people with AN exhibit high levels of emotion avoidance (Wildes et al., 2010; Hambrook et al., 2011; Westwood et al., 2017), social anhedonia (defined as an absence of pleasure derived from being with people) (Tchanturia et al., 2012), and difficulties in emotion regulation (Harrison et al., 2010; Racine and Wildes, 2013; Lavender et al., 2015). Additionally, experimental studies in AN have shown increased urges to engage in diets when experiencing emotions that they perceive as negative (Wildes et al., 2012). Collectively, these studies suggest that emotion avoidance, or the avoidance of experiencing and expressing emotions (Wildes et al., 2010) may be a characteristic feature of the way in which emotions are processed in people with AN.

Furthermore, in recent years a series of experimental studies have investigated the difficulties of expressing emotions in people with AN. A meta-analysis found that, when exposed to film clips eliciting emotions, participants with AN showed reduced spontaneous facial expression of both positive and negative emotion, compared to healthy controls (HC), with a large and medium effect size, respectively, Davies et al. (2016) and Leppanen et al. (2017). Additionally, there is preliminary evidence to suggest that participants with AN are less accurate than HC when deliberately attempting to show facial expressions of emotions (Dapelo et al., 2016).

Interestingly, findings suggest that individuals who have recovered from AN tend to express more positive emotions facially than those in the acute phase of the illness (Davies et al., 2013; Dapelo et al., 2016), which is in line with the hypothesis that these socioemotional difficulties contribute to the maintenance of the ED.

Besides its possible role as a perpetuating factor for AN, the avoidance of expressing emotions may impact the ability of individuals with AN to build and maintain social relationships, affecting their social functioning. For example, expressing positive emotions play a key role in establishing rapport and alliance in social interactions (Schmidt and Cohn, 2001), even expressing negative emotions, such as sadness or anger, may have positive effects on affiliation by promoting responsiveness and a sense of intimacy in social relationships (Fischer et al., 2008; Graham et al., 2008). Furthermore, studies on individuals with limited facial expression indicate that they are perceived as reserved and unhappy, and report being less interested in establishing friendship ties with them (Tickle-Degnen and Lyons, 2004; Hemmesch et al., 2009; Bogart et al., 2014). Individuals with AN have also reported feelings of isolation (Robinson et al., 2015), difficulties establishing friendships (Doris et al., 2014; Westwood et al., 2016; Sedgewick et al., 2019; Datta et al., 2021), and a negative impact of the disorder in the social arena (Tchanturia et al., 2013). Evidence within the literature suggests that emotion avoidance may be a contributing factor for the reported social difficulties in individuals with AN.

The social difficulties reported in the literature by people with AN, warrant the establishing for treatment strategies aimed at enhancing emotion expression. Cognitive remediation and emotion skills training (CREST) is a manualised treatment for inpatients with severe AN that was developed with the purpose of targeting emotional processing through psychoeducation and interactivity (Money et al., 2011; Tchanturia et al., 2014, 2015).

Research exploring the effects of CREST in people with AN shows that those who engaged in CREST exhibited a decrease in social anhedonia and in self-reported alexithymia, and provided positive qualitative feedback (Money et al., 2011; Tchanturia et al., 2014). CREST is still under development and there is continuous effort to improve outcomes by modifying its interventions based on research evidence and on patient’s feedback.

In this context, we designed a single-session workshop with the goal of raising awareness of the importance of enhancing positive communication and emotional expression in those receiving treatment for AN. The workshop was delivered in groups and lasted 90 min. We named the workshop as a “Positive Communication Workshop” to emphasize the positive, welcoming character of the intervention, rather than focusing on emotions *per se*, which could feel more threatening and anxiety-provoking for participants. The workshop included psychoeducation as well as activities designed to increase non-verbal expression of emotions and promoting eye gaze, and reflection. Details on the workshop content and activities are provided in the appendix number 1.

The present study aimed to pilot a single session workshop with the goal of raising awareness of the importance in enhancing positive communication and emotional expression in those receiving treatment for AN; whilst also learning from participants’ views about this workshop. Specifically, our aims were to: (1) explore the feasibility of positive communication workshops in ED inpatients’ and day services’ treatment programmes; (2) to evaluate feedback from patients; and (3) to plan future potential developments with positive communication workshops.

## 2. Methodology

### 2.1. Participants

A total of 41 participants took part in the workshop. All participants were female adults aged between 18 and 50 years old, with the mean age at 33 (SD = 12.2). Participants had a diagnosis of AN according to the DSM-5, were in various stages of their illness, and were receiving treatment through either an inpatient admission or day services at South London and Maudsley Specialist Eating Disorder Services. All participants were of consecutive admissions to clinical services. There were no exclusion criteria, everyone admitted to these services were invited to participate in the workshop. Study permission was obtained from the local research and governance committee at South London and Maudsley NHS Foundation Trust.

### 2.2. Self-report measures

All workshop participants were asked to complete 2 questionnaires, one before and one after the workshop. In addition, participants completed a feedback form at the end of the workshop. All the measures were designed by the investigators to obtain feedback from participants in every-day clinical practice:

#### 2.2.1. Pre-workshop questionnaire

The questionnaire was designed to enable participants to rate their understanding of their own communication styles prior to attending the workshop (Appendix 2). This pre-workshop questionnaire consisted of 5 questions: 1. “I enjoy social situations; 2. “I find social situations uncomfortable”; 3. “Making eye contact with people is difficult for me”; 4. “People say they cannot tell how I am feeling from my facial expressions”; and 5. “I tend not to use gestures when talking to people.” It used a 5-point Likert scale ranging from 1 (“Completely disagree”) to 5 (“Agree completely”).

#### 2.2.2. Post-workshop questionnaire

This post-workshop questionnaire had two groups of questions (Appendix 2). The first group of questions assessed the effectiveness of the workshop, questions included: 1. “How much did you enjoy the group?”; 2. “How useful was the group?”; 3. “Are you more aware of your communication styles as a result of the group?”; and 4, “How relevant was the group content to your communication?” The second group of questions assessed their understanding of the importance and their confidence in different communication styles including: eye contact; tone of voice; facial expression; and body language. This questionnaire also used a 5-point Likert scale ranging from 1 (“Not at all”) to 5 (“Very”).

#### 2.2.3. Feedback questionnaire

Feedback form with two open-ended questions asking participants what they liked most about the workshop and if they had any suggestions for improvements (Appendix 3).

### 2.3. Procedure

The workshop protocol was developed in collaboration with researchers and clinicians in the Eating Disorder Services at SLaM. The workshop consisted of three interactive activities, followed by a reflective component (Appendix 1).

After introducing themselves, workshop facilitators (*N* = 2) alternated in providing a brief overview of the experimental research about positive communication. This included an introduction on the importance of body language, tone of voice, and smiling as a social rewarding signal. Facilitators then shared current research findings on the poor use of positive emotional signaling in eating disorders followed by practical exercises. Psychoeducational materials in addition to these exercises were adapted from the actors training programmes by principal author (KT). Before they were included in the workshop format, these exercises were evaluated and calibrated for the purpose by KT and her research lab.

The session started with a psychoeducation section, in which the relevance of expressing emotions and communicating inner states for social functioning in the context of eating disorders was explained. This section was conducted in an interactive format, with the use of videos, and graphical images.

Then, three interactive activities were carried out, the first of which aimed to promote eye gaze. The other two activities encouraged participants to increase their expressivity using games involving the imitation of others’ facial emotional expressions, and non-verbal communication of positive emotions. Following this, the participants were given time to reflect and to relate the content of the interactive activities to the importance of communicating emotions in daily life.

The psychoeducation and interactive elements of this workshop were developed to make the workshop easier to engage with. Flyers were produced to invite patients under inpatient and day services care to attend these workshops. Pre- and post- workshop questionnaires were collected at the beginning and end of the workshop (Table 1). Participants of the workshop were also provided with information leaflets to refer to after the workshop.

**Table 1:** Exploratory statistics from pre-workshop questionnaire (*n* = 40).

Pre-workshop questions	Mean score	Std dev.	Mode	Min (Score of 1)	Max (score of 5)
“I enjoy social situations”	3.1	1.1	2 and 4 (*n* = 12; 29.3%)	4.9% (*n* = 2)	7.3% (*n* = 3)
“I find social situations uncomfortable”	3.2	1.2	4 (*n* = 13; 31.7%)	12.2% (*n* = 5)	12.2% (*n* = 5)
“Making eye contact is difficult for me”	2.9	1.4	2 (*n* = 11; 26.8%)	19.5% (*n* = 8)	14.6% (*n* = 6)
“People say they cannot tell how I am feeling from my facial expression”	2.7	1.3	2 (*n* = 11; 31.7%)	22.0% (*n* = 9)	9.8% (*n* = 4)
“I tend not to use gestures when talking to people”	2.5	1.3	1 and 2 (*n* = 11; 26.6%)	26.6% (*n* = 11)	7.3% (*n* = 3)

### 2.4. Data analysis

Feedback provided in the pre- and post-workshop questionnaires was analysed using the statistical software IBM SPSS Statistics ver. 27, with frequencies and exploratory statistics (means, SD, min, max and mode). The post-workshop questionnaires (Table 2) were analysed using a paired samples *t*-test.

**Table 2:** Exploratory statistics from post-workshop questionnaire, comparing participants’ understanding of the importance and confidence in using positive communication strategies (*n* = 37).

Post-workshop questions: Importance and confidence in using positive communication strategies		Mean score	Std dev.	Effect size of difference between importance and confidence
Eye contact	Importance	4.6	0.6	
	Confidence	3.0	1.2	1.4
Tone of voice	Importance	4.7	0.5	
	Confidence	3.6	0.9	1.0
Facial expression	Importance	4.5	0.7	
	Confidence	3.4	1.2	1.2
Body language	Importance	4.6	0.5	
	Confidence	2.8	1.2	1.7

Cohen *d* effect sizes (>0.8 is considered a large effect size).

Qualitative data was obtained from the participants’ responses on the following questions in the feedback questionnaire: “What did you like most about the group,” and “Ideas for improvements.” Two researchers independently identified themes from these responses before comparing and agreeing themes identified in their meeting. This data was then analysed using inductive thematic analysis.

## 3. Results

In total, 41 patients attended a positive communication workshop and completed questionnaires. Four of these participants had incomplete data as they had returned completed post-workshop questionnaires. There were also ten other participants who attended the workshops, however, they only expressed their feedback verbally, and so this was not included in the analysis to keep the report consistent.

### 3.1. Quantitative feedback from participants

The results show in the post-workshop questionnaires, that participants rated highly the relevance and usefulness of the communication styles demonstrated in the workshop (Table 3). Participants also rated the importance of using positive communication strategies higher than their confidence to use them with a large effect size on all domains (Table 2).

**Table 3:** Exploratory statistics from the post-workshop questionnaire (*n* = 37).

Post-workshop questions: Effectiveness of the positive communication workshop	Mean score	Std dev.	Mode	Min (Score of 1)	Max (score of 5)
How much did you enjoy the group?	3.7	1.0	3 (*n* = 14; 34.1%)	0% (*n* = 0)	24.4% (*n* = 10)
How useful was the group?	3.7	1.0	3 (*n* = 16; 39%)	2.7% (*n* = 1)	29.2% (*n* = 12)
Are you more aware of your communication style, as a result of the group?	3.8	1.2	4 (*n* = 13; 31.7%)	4.9% (*n* = 2)	29.3% (*n* = 12)
How relevant was the group content to your communication?	3.9	0.9	4 (*n* = 14; 34.1%)	0% (*n* = 0)	26.8% (*n* = 11)

### 3.2. Qualitative feedback from participants

Qualitative feedback was collected using open-ended questions in both inpatient and day services programmes in order to improve future workshop content and delivery. Overall, both written and verbal feedback was very positive. Inductive thematic analysis was used to identify themes in the comments provided. Three key themes were identified for both questions and are summarized below (examples of quotes for each theme are in given in Tables 4, 5):

**Table 4:** A table of participant quotes providing examples for each theme.

Theme	Participant examples
Activities	“Learning from basic examples and how powerful it is” “Interactive exercises we did really highlighted the need for positive communication” “The tasks, the videos and the way everything was explained” “Interactive exercised to illustrate theory” “Fun, game-based exercises, not too serious but still informative” “I enjoyed the interactive elements where we were putting into practice what we were learning” “I liked the interactive games and parts. It was great to get hands on and see communication in action” “Variety of activities”
Facilitators	“[Facilitators] were welcoming to the group which encouraged conversation and discussion” “It felt collaborative” “[Facilitator] was very passionate about everything” “Welcoming, friendly and fun atmosphere” “[Facilitator] was nice and kind, very engaging with the group” “I really liked the open, balanced, curious yet well-informed stance that was taken by the group facilitators” “[Facilitators] did such a great job in making us feel comfortable to be open” “The leaders kept it buoyant”
Relevance	“I found it interesting and helpful to my situation” “Relevant to issues I face and made me aware of my own communication” “It made me realize how my actions-facial expressions, emotions etc. make other people feel and how to improve these” “Systematically broke down myths I have never questioned” “Learning how to apply different ways of thinking to my life and how implementing a more positive mindset could benefit other aspects of my life”

**Table 5:** A table of participant quotes providing examples for each theme.

Theme	Participant examples
Amount of content	“Could have been longer” “Need more time as there is a lot of material” “Not as much to get through in one session” “More sessions and regularly run the group” “I feel the activities would be better split throughout the full 1.5-h session rather than all at the end” “The group games could feature even more – I loved these” “More group activities”
Continued learning	“Spend more time on developing strategies for positive thinking – more personal” “Perhaps give handouts to carry forward” “It would be useful to be told how to develop out own communication skills and be more assertive” “tips” “More focus on tips to improve communication in more in depth ways”
Participation	“Might be good to leave the question opens, say to put it in a feedback form” “People might feel nervous” “Make it compulsory to be attending but not participating” “A lot of people are quite shy with eating disorders”

#### 3.2.1. What I liked the most

##### 3.2.1.1. Activities

Participants expressed that the activities in the workshop demonstrating and practicing different positive communication strategies were fun, engaging and interactive.

##### 3.2.1.2. Facilitators

Participants reported that the group facilitators were welcoming, kind, and friendly.

##### 3.2.1.3. Relevance

Participants commented that the psychoeducational materials were useful, informative, relevant, and that the content of the workshop was interesting.

#### 3.2.2. Ideas for improvements

##### 3.2.2.1. Amount of content

Participants commented that there was considerable amount of content to cover in the time given for the workshop. Suggestions were made to either include less content, allow more time or to find a better balance of content and activities.

##### 3.2.2.2. Continued learning

Participants expressed that they could have benefitted from more time and practice on developing these strategies outside of the workshop and would have liked more materials that they could take away with them.

##### 3.2.2.3. Participation

Participants reported they would have preferred participation in discussions and activities to have been optional rather than expected throughout the workshop.

In total, 80% of the workshop participants returned completed questionnaires and the feedback was largely positive (Table 4). There were a number of suggestions for improvements to the workshop including having more time, more interactive games and group activities and further detailed handouts to provide post-workshop (Table 5).

## 4. Discussion

The data from this study, along with previous research demonstrating positive patient experience and clinical outcomes in group therapies (Sparrow and Tchanturia, 2016), indicated that there is feasibility for group workshops to be delivered as add-ons in the ED treatment programme.

Findings from the pre-workshop questionnaire suggest that the patients attending the workshop were aware of their difficulties in social interactions, such as finding social situations difficult and having problems with eye contact, expressing emotions and engaging in social communication. This is in line with findings from experimental studies demonstrating that individuals with AN show significantly less eye contact than healthy controls (Harrison et al., 2018), they also spend less time watching social stimuli (Kerr-Gaffney et al., 2022). As expected, we found a large effect size difference in participant’s self-reported ratings of the importance and their confidence in using eye contact, tone of voice, body language in the communication; suggesting that participants found all key elements of effective communication important but were not confident using this knowledge in real life situations. The results from the post-workshop questionnaire indicate that patients found the workshop content relevant, useful, and were more aware of their communication style as a result.

The positive feedback elicited from participants on the feedback questionnaires highlights the acceptability of the positive communication group workshop. Participants generally found the group experience positive, and feedback from the workshop indicated that the majority of participants found it helpful. They particularly enjoyed the interactive and easy nature of the workshop, in addition to learning about different positive communication strategies and how they have an impact on their lives. The positive feedback and acceptability of the intervention suggests that developing these workshops further has potential.

The feedback obtained in the post-workshop questionnaires also suggested further improvements that could be made to enhance the workshop. In the themes that emerged, there would be merit to explore, in future workshops, increasing the time of the session and developing the take-away materials to allow maximum benefit of the content. It would also be interesting to examine the differences in outcomes and feedback of these potential workshop developments with the current evaluation.

The idea of further opportunities post-workshop to practice positive communication skills is an interesting one. Individuals with AN receiving inpatients or day services treatment will often remain isolated and avoid contact with other patients, thus, it could be worthwhile for services to observe participants’ use of the skills at post-workshop. This could be in the form of follow-up opportunities with workshop participants within clinical services to evaluate the use of the learned skills in everyday life and provide further support.

Moreover, it would be interesting to assess the usefulness of carrying out this workshop in younger patients. Even though there is evidence that social anxiety increases with age (Kerr-Gaffney et al., 2018) it is relevant to note that social anxiety and interpersonal difficulties usually begin before the onset of the eating disorder and can predispose to the development of AN (Treasure et al., 2020). Therefore, providing tools to enhance positive communication and emotional expression early on to adolescent patients with AN might have a positive impact, preventing these social difficulties from becoming more severe.

This pilot study has some strengths worth mentioning, it is the first case series of its kind, reporting on pilot work with positive communication workshops. It contains a protocol allowing others to replicate and develop the workshop and has allowed us to suggest improvements to the existing protocol, which paves the way for these workshops to be trialed in larger studies. This pilot study aimed to explore the feasibility of the one-off positive communication workshops and subjective outcomes and concludes acceptability and feasibility of these workshops, meeting objectives 1 and 2. Follow-up studies would benefit from more objective evaluation based on investigator-administered scales of communication styles and other variables.

The group intervention design of the workshop is another strength. Psychological group interventions can generally bring unique benefits that are not achievable when working individually with patients. These benefits include sharing experiences and learning from others in a safe and therapeutic environment, being with other people and practicing interpersonal skills. Individuals with AN have difficulties with social contacts, and report high levels of social anhedonia (Harrison et al., 2014; Kerr-Gaffney et al., 2018). It has been observed that patients with AN often remain isolated and tend to avoid communicating with other patients in the intensive treatment programmes (Hambrook et al., 2011; Tchanturia et al., 2012; Doris et al., 2014; Westwood et al., 2016). Therefore, by employing a group design for the workshop, these benefits are promoted.

In terms of limitations, we note that there is an absence of a control group in this evaluation. Future studies would benefit from including a control group, larger numbers of participants and additional measures to capture change before and after the intervention in comparison to healthy controls. For specificity, this study only included patients with an AN diagnosis, and it would be valuable to investigate other ED diagnoses and comorbidities in future studies. It will be important for future studies to have clarity and analyse subgroups with and without an autism comorbidity to explore the question of the similarities and differences between these groups in response to treatment.

## 5. Conclusion

Positive communication workshops seem to be a feasible format for patients with AN. This pilot demonstrated that the workshop was able to enhance patients’ awareness of their communication and gave opportunity to learn strategies to enhance their confidence in managing social communication. Improving awareness of communication styles may help participants to manage distress and form healthy coping mechanisms, further supporting their recovery from EDs and understand their social interaction style.

## Data availability statement

The datasets presented in this article are not readily available because as they contain information that could compromise the privacy of the participants. Requests to access the datasets should be directed to Kate.tchanturia@kcl.ac.uk.

## Ethics statement

The studies involving humans were approved by the South London and Maudsley Clinical governance board (2009/09). The studies were conducted in accordance with the local legislation and institutional requirements. Written informed consent for participation was not required from the participants or the participants’ legal guardians/next of kin in accordance with the national legislation and the institutional requirements. All participants were age 18 and above and consented to publish the data anonymously.

## Author contributions

KT and MD: conceptualization. KT: methodology and supervision. PC: formal analysis, VH and PC: investigation. KT, PC, and VH: data curation. KT, PC, VH, and MD: writing review and editing. JW, PC, and VH: project administration. All authors have read and agreed to the submitted version of the manuscript.

## Funding

KT received the funding from UKRI Medical Research Council (MRC-MRF) Fund [MR/R004595/1].

## Conflict of interest

The authors declare that the research was conducted in the absence of any commercial or financial relationships that could be construed as a potential conflict of interest.

## Publisher’s note

All claims expressed in this article are solely those of the authors and do not necessarily represent those of their affiliated organizations, or those of the publisher, the editors and the reviewers. Any product that may be evaluated in this article, or claim that may be made by its manufacturer, is not guaranteed or endorsed by the publisher.
