# Supplementary material for: Positive communication workshops: are they useful for treatment programmes for anorexia nervosa?
Source: Front Psychol. 2023 Aug 14;14:1234928. doi: 10.3389/fpsyg.2023.1234928 (PMC10461805; doi:10.3389/fpsyg.2023.1234928)
Supplement: Supplementary file 1 [file Data_Sheet_1.PDF]

Supplementary Material

Positive Communication Workshops: Are they useful for treatment programmes for Anorexia Nervosa?

Kate Tchanturia[1,2,3*], Phillippa Croft[2], Victoria Holetic[2], Jessica Webb[2], Marcela Marin Dapelo[1]

[1]Institute of Psychiatry, Psychology and Neuroscience, Department of Psychological Medicine, King's College London, London, UK

[2]Eating Disorders National Service, South London and Maudsley NHS Foundation Trust, London, UK

[3]Ilia State University, Georgia

*** Correspondence:**
Professor Kate Tchanturia, PO59, Institute of Psychiatry, Psychology and Neuroscience, Department of Psychological Medicine, King's College London, De Crespigny Park, Denmark Hill, London, SE5 8AF, UK.
Tel: +44 (0)207 848 0134, Fax: +44 (0)207 848 0182, Email: kate.tchanturia@kcl.ac.uk

1 Supplementary tables

1.1 Table 1. Exploratory statistics from pre-workshop questionnaire (n=40).

**Pre-workshop questions**	**Mean score**	**Std dev.**	**Mode**	**Min (Score of 1)**	**Max (score of 5)**
"I enjoy social situations"	3.1	1.1	2 and 4 (n= 12; 29.3%)	4.9% (n=2)	7.3% (n=3)
"I find social situations uncomfortable"	3.2	1.2	4 (n= 13; 31.7%)	12.2% (n=5)	12.2% (n=5)
"Making eye contact is difficult for me"	2.9	1.4	2 (n=11; 26.8%)	19.5% (n=8)	14.6% (n=6)

"People say they can't tell how I am feeling from my facial expression"	2.7	1.3	2 (n=11; 31.7%)	22.0% (n=9)	9.8% (n=4)
"I tend not to use gestures when talking to people"	2.5	1.3	1 and 2 (n=11; 26.6%)	26.6% (n=11)	7.3% (n=3)

1.2 Table 2. Exploratory statistics from the post-workshop questionnaire (n=37).

**Post-workshop questions:** Effectiveness of the positive communication workshop.	**Mean score**	**Std dev.**	**Mode**	**Min (Score of 1)**	**Max (score of 5)**
How much did you enjoy the group?	3.7	1.0	3 (n=14; 34.1%)	0% (n=0)	24.4% (n=10)
How useful was the group?	3.7	1.0	3 (n=16; 39%)	2.7% (n=1)	29.2% (n=12)
Are you more aware of your communication style, as a result of the group?	3.8	1.2	4 (n=13; 31.7%)	4.9% (n=2)	29.3% (n=12)
How relevant was the group content to your communication?	3.9	0.9	4 (n=14; 34.1%)	0% (n=0)	26.8% (n=11)

1.3 Table 3. Exploratory statistics from post-workshop questionnaire, comparing participants' understanding of the importance and confidence in using positive communication strategies (n=37).

**Post-workshop questions:** Importance and confidence in using positive communication strategies.		**Mean score**	**Std dev.**	**Effect size of difference between importance and confidence**
Eye contact	Importance	4.6	0.6	
	Confidence	3.0	1.2	**1.4**
Tone of voice	Importance	4.7	0.5	
	Confidence	3.6	0.9	**1.0**
Facial expression	Importance	4.5	0.7	
	Confidence	3.4	1.2	**1.2**
Body language	Importance	4.6	0.5	
	Confidence	2.8	1.2	**1.7**

Cohen d effect sizes (>0.8 is considered a large effect size).

1.4 Table 4. A table of participant quotes providing examples for each theme.

**Theme**	**Participant Examples**
Activities	"Learning from basic examples and how powerful it is"<br><br>"Interactive exercises we did really highlighted the need for positive communication"<br><br>"The tasks, the videos and the way everything was explained"<br><br>"Interactive exercised to illustrate theory"

	"Fun, game-based exercises, not too serious but still informative"<br><br>"I enjoyed the interactive elements where we were putting into practice what we were learning"<br><br>"I liked the interactive games and parts. It was great to get hands on and see communication in action"<br><br>"Variety of activities"
Facilitators	"[Facilitators] were welcoming to the group which encouraged conversation and discussion"<br><br>"It felt collaborative"<br><br>"[Facilitator] was very passionate about everything"<br><br>"Welcoming, friendly and fun atmosphere"<br><br>"[Facilitator] was nice and kind, very engaging with the group"<br><br>"I really liked the open, balanced, curious yet well-informed stance that was taken by the group facilitators"<br><br>"[Facilitators] did such a great job in making us feel comfortable to be open"<br><br>"The leaders kept it buoyant"
Relevance	"I found it interesting and helpful to my situation"<br><br>"Relevant to issues I face and made me aware of my own communication"<br><br>"It made me realise how my actions-facial expressions, emotions etc. make other people feel and how to improve these"<br><br>"Systematically broke down myths I have never questioned"<br><br>"Learning how to apply different ways of thinking to my life and how implementing a more positive mindset could benefit other aspects of my life"

1.5 Table 5. A table of participant quotes providing examples for each theme.

**Theme**	**Participant examples**
Amount of content	"Could have been longer"

	"Need more time as there is a lot of material"<br><br>"Not as much to get through in one session"<br><br>"More sessions and regularly run the group"<br><br>"I feel the activities would be better split throughout the full 1.5-hour session rather than all at the end"<br><br>"The group games could feature even more – I loved these"<br><br>"More group activities"
Continued learning	"Spend more time on developing strategies for positive thinking – more personal"<br><br>"Perhaps give handouts to carry forward"<br><br>"It would be useful to be told how to develop out own communication skills and be more assertive"<br><br>"tips"<br><br>"More focus on tips to improve communication in more in depth ways"
Participation	"Might be good to leave the question opens, say to put it in a feedback form"<br><br>"People might feel nervous"<br><br>"Make it compulsory to be attending but not participating"<br><br>"A lot of people are quite shy with eating disorders"

2 Appendices

2.1 Appendix 1. Workshop summary table

Name of activity	Aim of the activity	Description	Materials needed	Time
Psychoeducation	To inform about relevance of positive emotion	Presentation and video showing evidence-based	1. Laptop<br>2. Presentation	15 minutes

	and social communication for socioemotional functioning	information about positive communication	3. Face still paradigm video	
Positive treasure	To raise awareness of resources/things that make people feel good/pleasure	Participants make a mind-map identifying things that make them feel good/pleasure	1. Paper<br>2. Pencils	5 minutes
Zip Zap Boing	To warm up and get participants ready for more active exercises	Participants need to clap saying “Zip”, “Zap”, or “Boing” and the word being said determines which participant goes next	None	3 minutes
Rehearsing muscles	To show how to warm up our body, facial muscles and body	The clinician will show participants techniques that actors use to warm up in rehearsals	None	4 minutes
Masks	To show how body language	The clinician or a volunteer puts a mask over her face, choses an	1. Mask<br>2. Emotion words	5 minutes

	can communicate emotional states	emotion card and "acts out" the emotion using body language. Participants need to guess what emotion is being depicted		
Count to 20	To show the relevance of eye gazing in social interaction	Participants need to coordinate to be able to count to 20 as a group. The first time they do it looking at their shoe. The second time, looking at people's eye	None	8 minutes
*Mirror**	To exercise mirroring others body movements	Two people stand facing each other and one tries to reflect the other's movements as accurately as possible. Then they switch	None	5 minutes
*Shake hands**	To show the importance of listening to others	Participants walk around, when they get a signal they need to find	None	5 minutes

		someone, shake hands and introduce themselves disclosing something about them. Then people need to introduce the other person		
Reflection	To reflect about how this exercises can be translated into real life	Discussion	1. Handouts with tips	5 minutes
**TOTAL**				50 minutes

***only one**, either Mirror or Shake hands. This is optional; we had one hour and half for the workshops to make sure we had time for discussion; extra time for questions and pace of the group.

2.2 Appendix 2. Positive communication workshop pre-group questionnaire

Positive communication pre-group questionnaire

Please indicate the extent to which you agree with each statement by circling the number from 1 to 10.

1 = completely disagree

5 = agree completely

I enjoy social situations

1	2	3	4	5

I find social situations uncomfortable

1	2	3	4	5

Making eye contact with people is difficult for me

1	2	3	4	5

People say they can't tell how I am feeling from my facial expression

1	2	3	4	5

I tend not to use gestures when talking to people

1	2	3	4	5

2.3 Appendix 3. Positive communication workshop post-group questionnaire

<u>Group Feedback Questionnaire – Positive Communication</u>

1.) How much did you enjoy the group?

Did not enjoy at all		quite enjoyed it		really enjoyed
**1**	**2**	**3**	**4**	**5**

2.) How useful was the group?

Not at all useful		quite useful		really useful
**1**	**2**	**3**	**4**	**5**

3.) Are you more aware of your communication style, as a result of the group?

No, not at all		a bit		yes, lots
**1**	**2**	**3**	**4**	**5**

4.) How relevant was the group content to your communication?

Not at all relevant		some relevance		very relevant
**1**	**2**	**3**	**4**	**5**

5.) How important do you think the following aspects are for positive communication?
(1 = not important at all; 5 = extremely important)

**Eye contact**	**1**	**2**	**3**	**4**	**5**
**Tone of voice**	**1**	**2**	**3**	**4**	**5**

**Facial expression**	**1**	**2**	**3**	**4**	**5**
**Body language**	**1**	**2**	**3**	**4**	**5**

6.) How confident do you feel about using the following aspects in social situations? (1 = not at all confident; 5 = very confident)

**Eye contact**	**1**	**2**	**3**	**4**	**5**
**Tone of voice**	**1**	**2**	**3**	**4**	**5**
**Facial expression**	**1**	**2**	**3**	**4**	**5**
**Body language**	**1**	**2**	**3**	**4**	**5**

7.) What did you like the most about the group?

…………………………………………………………………………………………………
…………………………………………………………………………………………………
…………………………………………………………………………………………………
……………………………………………………………………………...
....

8.) Please give your ideas about how we could improve the group in the future:

…………………………………………………………………………………………………
…………………………………………………………………………………………………
…………………………………………………………………………………………………
……………………………………………………………………………...
....

9.) What other groups have you attended on the ward?

……………………………………………………………………………………………………

……………………………………………………………………………………………………

……………………………………………………………………………………………………

…………………………………………………………………..

....
